# Dried Plum’s Polyphenolic Compounds and Carbohydrates Contribute to Its Osteoprotective Effects and Exhibit Prebiotic Activity in Estrogen Deficient C57BL/6 Mice

**DOI:** 10.3390/nu14091685

**Published:** 2022-04-19

**Authors:** Brenda J. Smith, Bethany Hatter, Karley Washburn, Jennifer Graef-Downard, Babajide A. Ojo, Guadalupe Davila El-Rassi, Robert H. Cichewicz, Mark Payton, Edralin A. Lucas

**Affiliations:** 1 Department of Nutritional Sciences, Oklahoma State University, Stillwater, OK 74078, USA; bhatter@ostatemail.okstate.edu (B.H.); karleyw@ostatemail.okstate.edu (K.W.); official.babajo@gmail.com (B.A.O.); edralin.a.lucas@okstate.edu (E.A.L.); 2 Department of Nutritional Sciences, University of Oklahoma Health Sciences Center, Oklahoma City, OK 73117, USA; jennifer-graefdownard@ouhsc.edu; 3 Department of Food and Animal Sciences, Oklahoma State University, Stillwater, OK 74078, USA; guadalupe.davila_de_el_rassi@okstate.edu; 4 Department of Chemistry and Biochemistry, University of Oklahoma, Norman, OK 73019, USA; rhcichewicz@ou.edu; 5 Department of Biomedical Sciences, Rocky Vista University, Parker, CO 80134, USA; mpayton@rvu.edu

**Keywords:** bone, polyphenols, dried plum, menopause, osteoporosis, short chain fatty acids, gut-bone axis

## Abstract

Evidence of dried plum’s benefits on bone continues to emerge. This study investigated the contribution of the fruit’s polyphenol (PP) and carbohydrate (CHO) components on a bone model of postmenopausal osteoporosis to explore their prebiotic activity. Osteopenic ovariectomized mice were fed diets supplemented with dried plum, a crude extract of dried plum’s polyphenolic compounds, or the PP or CHO fraction of the crude extract. The effects of treatments on the bone phenotype were assessed at 5 and 10 weeks as well as the prebiotic activity of the different components of dried plum. Both the CHO and PP fractions of the extract contributed to the effects on bone with the CHO suppressing bone formation and resorption, and the PP temporally down-regulating formation. The PP and CHO components also altered the gut microbiota and cecal short chain fatty acids. These findings demonstrate that the CHO as well as the PP components of dried plum have potential prebiotic activity, but they have differential roles in mediating the alterations in bone formation and resorption that protect bone in estrogen deficiency.

## 1. Introduction

Research focused on dietary strategies to optimize bone health and reduce life-long risk of osteoporotic fractures has resulted in the investigation of a wide range of foods and their bioactive components [1,2,3]. One class of foods that has received considerable attention from researchers in the field are the dark red and purple stone fruits, some of which are a good source of phenolic acids (i.e., hydroxycinnamic acids) [4,5]. Dysregulation of oxidative homeostasis and immune cell function are central to the etiology of bone loss that occurs with estrogen deficiency as well as aging [6,7]. Thus, it stands to reason that incorporating such fruits with antioxidant and anti-inflammatory compounds into the diet could convey osteoprotective effects on the skeleton.

The dried plum (*Prunus domestica* L.) is one of the fruits in this class that has been studied extensively in both clinical and pre-clinical studies. Initial studies in postmenopausal women demonstrated the efficacy of dried plum (100 g/d) at improving bone biomarkers and protecting against the decline in spine and forearm BMD [8,9]. Subsequently, a 50 g/d dose of dried plum was as effective as 100 g/d at preserving bone in another study of postmenopausal women [10]. More recently, studies with older men showed that dietary supplementation with dried plum (100 g/d) reduced serum osteocalcin at 3 months and bone resorption markers (i.e., tartrate-resistant acid phosphatase-5b or TRAP5b and C-terminal collagen cross-link or CTX) at 6- and 12-months, but no improvements in BMD [11,12]. Pre-clinical studies have revealed that dried plum attenuates bone loss in animal models of gonadal hormone deficiency, rheumatoid arthritis, ionizing radiation, and aging, and also restores both trabecular and cortical bone in osteopenic animal models [13,14,15,16,17]. The fruit’s anti-inflammatory properties have been reported in clinical studies with postmenopausal women (50 g/d) to decrease interleukin (IL)-6 and tumor necrosis factor (TNF)-*α*, after 6 months [18]. Likewise, dried plum suppressed T cell activation in the ovariectomized mouse model in ex vivo experiments [19]. In vitro studies have demonstrated the ability of the fruit’s polyphenols to down-regulate the mitogen activated protein kinase (MAPK) pathway’s ability to promote osteoclast differentiation through Nfatc1, and osteoclast activity via the expression of matrix metalloproteinase (MMP)9 and cathepsin K [17,20]. Further, these polyphenolic compounds restore osteoblast activity that has been suppressed by TNF-α and can normalize bone morphogenetic protein (BMP)-2 and transforming growth factor β signaling [21]. The antioxidant capacity of the whole fruit and its polyphenolic compounds has also been reported in human subjects, animal models and in vitro systems [15,18,22,23]. Although these studies support the bone protective, anti-inflammatory, and antioxidant properties of dried plums, the relatively low bioaccessibility of the fruit’s polyphenols has brought into question the active components and the target(s) of their activity [24,25,26]. 

Our laboratory previously reported that a crude extract of dried plum’s polyphenols restored bone in an aged, osteopenic ovariectomized rat model [27]. However, some carbohydrates were retained in the extract in conjunction with the polyphenolic compounds, leaving the relative contribution of these two components unclear. Evidence that the gastrointestinal tract and the microbiota that reside therein are potential targets for interventions using prebiotic and probiotics has rapidly emerged in the bone field as it has in many other fields of biomedical research. Studies designed to understand the gut–bone axis have revealed several new promising lines of research focused on gut-derived hormones and secondary metabolites, the integrity of the gut barrier, as well as the trafficking of gut mucosal immune cells (e.g., T regulatory cells) to bone [28,29,30]. Thus, this study aimed to determine the extent to which the polyphenolic (PP) compounds and carbohydrates (CHO) restore bone in an osteopenic, ovarian hormone deficient mouse model. Furthermore, we explored how these components of dried plum alter bone metabolism and their potential prebiotic activity as demonstrated by alterations in the gut microbiota and cecal short chain fatty acids (SCFAs). We hypothesized that the PP fraction of the crude extract would have a greater effect on bone over time despite some potential prebiotic activity with the CHO fraction. Our results show that both the polyphenolic compounds and carbohydrates contribute to improved trabecular and cortical bone microarchitecture, however, their structural and metabolic effects on bone differ over time. Furthermore, both components appear to have prebiotic activity characterized by alterations in the microbiota and increases in SCFAs.

## 2. Materials and Methods

### 2.1. Animal Care and Experimental Design 

All procedures associated with this study adhered to guidelines set forth by the Oklahoma State University Animal Care and Use Committee. Three-month old ovariectomized (OVX) or sham-operated (Sham) C57BL/6 mice (Charles River) arrived at the Laboratory Animal Research facility at Oklahoma State University 7 days post-surgery and were acclimated for another 7 days prior to the initiation of the dietary treatments. This two-week post-surgery interval allowed OVX mice to lose bone prior to the start of treatment. 

**Figure 1:**
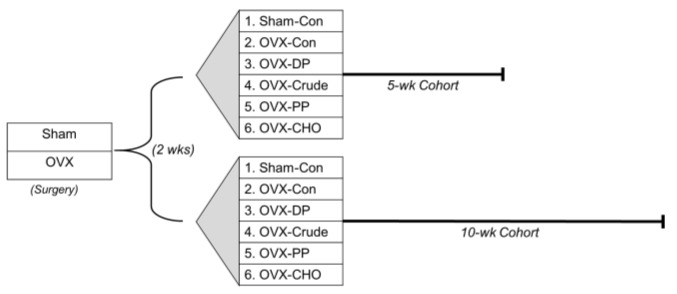
A schematic representation of the experimental design showing 2 wks post-surgery, sham-operated (Sham) or ovariectomized (OVX) C57BL/6 mice were randomly assigned to five different dietary treatments in a 5- or 10-week cohort of (*n* = 12 mice/group/cohort). Sham and OVX mice (Groups 1–4) made up the randomized block design and data were analyzed using a 1-way ANOVA. OVX mice in Groups 2, 4–6 made up the embedded 2 × 2 factorial that allowed for investigating the simple effects of the polyphenol (PP) and carbohydrate (CHO) fractions of the crude extract alone and in combination.

Sham and OVX mice were then randomly assigned to one of five diets and allocated to the 5- or 10-week treatment cohorts (Figure 1): control (AIN-93M; Con), or Con diet supplemented with dried plum (25% *w*/*w*; DP) or the crude extract (Crude) with a dose of polyphenols comparable to that in the DP (total phenolics = 2.975 gallic acid equivalent, GAE/g diet). The rationale for the two time points was to assess a shorter and longer-term response to treatments due to the time for alterations in the gut microbiota to occur and to account for differences in bone remodeling that occur over time with ovarian hormone deficiency. Two groups of sham-operated mice were maintained on the Con diet for 5 or 10 weeks. 

To investigate the contributions of the polyphenols and carbohydrates in the crude extract on bone, two additional dietary treatments were included in the 5- and 10-week cohorts consisting of the Con diet supplemented with a PP-rich or CHO-rich fraction (preparation described in Section 2.2 below) matched to the crude extract. These groups allowed us to embed a 2 × 2 factorial design to investigate the effects of PP and CHO alone and in combination while minimizing the need for additional mice. The PP and CHO from the individual fractions were each matched to the crude extract (total phenolics = 2.975 GAE/g diet and total carbohydrates = 131.1 g/kg diet). All diets were formulated to have similar total carbohydrate, fat, protein, fiber, calcium, phosphorus, potassium, and vitamin K content, and were based on the AIN-93M diet as previously described [27]. Throughout the study, food intake was monitored, mice were weighed weekly, and reverse osmosis water was provided ad libitum. 

At the end of the 5- and 10-week treatment periods, mice were fasted for 3 h, anesthetized (100 mg ketamine/10 mg xylazine) and whole-body DXA scans were performed to assess bone mineral content (BMC), area (BMA), and density (BMD) using PIXImus Series Software (Madison, WI, USA). Blood was collected via the carotid artery for bone biomarkers, followed by the collection of the bones and cecal contents. Uterine weight was recorded to verify OVX status and as an indicator estrogenic activity with the treatments. 

### 2.2. Preparation of the Polyphenol and Carbohydrate Fractions

Dried plum powder (‘Improved French’), provided by the CA Dried Plum Board, was used to prepare the crude extract and fractions. Dried plum powder (5 kg) was suspended in 80% methanol (1 L/kg powder) for 3 h, sonicated, and then filtered into a 20 L round bottom flask containing 3 kg of HP-20 resin. Filtered solids were rinsed (1 L of 80% methanol) into the same round bottom and the extract was dried to a slurry. This procedure was repeated 3×, overnight in the dark and all extracts were then combined. Drying the crude extract was accomplished by adding 2 × 500 mL of nanopure water to remove any residual methanol. The crude extract was subjected to rotary evaporation until a consistency of moist sand. Between each step, the extract was kept in a cold room to limit sample degradation. 

Next, the crude extract was subjected to chromatography (Diaion HP-20SS resin) and the CHO fraction followed by the PP fraction were eluted as described below. The removal of the CHO fraction was initiated by adding 2 L of deionized water to the crude extract and rinsing over cheesecloth into a vacuum flask. Rinses were performed 6× with 2 L each. The collected CHO fraction was flowed over 1 kg of fresh HP-20 resin to remove any residual phenolics. The CHO was dried until a syrup like consistency and stored in a large brown bottle at 4 °C. The mixture was subjected to freeze-drying and ground into a powder to be incorporated into the diets. 

The phenolic fraction (PP) was obtained from the resin by rinsing with 2 L 100% methanol 5×, allowing to sit for 10 min each time. This produced a dark extract which was dried, yielding a liquid with a syrup-like consistency and stored at 4 °C. The extract was subjected to freeze-drying, ground into a powder and sent for analyses. All test products used in this study were prepared by the Institute of Natural Products and Research Technologies (INPART) at the University of Oklahoma.

An aliquot of the dried plum powder, crude extract, and PP- and CHO-rich fractions sample was subjected to total phenolic analyses using the Folin–Ciocalteu assay [31]. The total phenolic content of the PP fraction was 54.91 GAE/g, but phenolics were not detectable in the CHO fraction. 

### 2.3. Micro-Computed Tomography (MicroCT) Analyses 

Evaluation of the trabecular and cortical bone was performed using micro-computed X-ray tomography (μCT40, SCANCO Medical, Brüttisellen, Switzerland). For trabecular bone analysis, secondary spongiosa within the distal femur metaphysis and the 5th lumbar vertebral body were analyzed as previously described [23]. At each site, trabecular bone morphometric parameters, including trabecular bone volume per unit of total volume (BV/TV), trabecular number (TbN), trabecular thickness (TbTh), and trabecular separation (TbSp) were evaluated. The femur mid-diaphysis was scanned to evaluate cortical bone microarchitecture. Cortical bone parameters included cortical thickness and area, medullary area and porosity. 

### 2.4. Serum Bone Biomarkers 

Serum bone biochemical markers were assessed using commercially available ELISAs (Immunodiagnostic Systems Inc., Gaithersburg, MD, USA) on samples collected at the 5- and 10-week study endpoint. Precollagen type 1 N-terminal propetide (P1NP), the precollagen type 1 N-terminal peptide that is cleaved during bone formation, was used to assess osteoblast activity. Tartrate-resitant acid phosphate (TRAcP) 5b, the metalloenzyme produced by osteoclasts during matrix degradation, was used as indicator of osteoclast activity or bone resorption. 

### 2.5. Short Chain Fatty Acids

Cecal samples were collected and weighed. SCFA concentrations of the cecal contents were assessed as previously reported [32] by suspending samples in ice-cold Millipore H_2_O, which was then spiked with the internal standard (1 mM 2-ethylbutyric acid in 12% formic acid). The pH of each sample was adjusted (pH = 2–3) using 5 M HCl and the samples were homogenized for 1 min. Supernatants were collected and filtered using a 0.45 mm polytetrafluoroethylene syringe filter (Agilent Technologies, Santa Clara, CA, USA). Gas chromatographic analysis was performed (Agilent 6890N GC system) with a flame ionizable detector and an automated sampler. The SCFA concentration was determined using a 5-point calibration curve, with standards from Sigma-Aldrich. The total amount of each cecal SCFAs, acetate (C 2:0), propionate (C 3:0), isobutyrate (C 4:0 i), butyrate (C 4:0 n), isovalerate (C 5:0 i) and valerate (C 5:0 n) was expressed in mM. 

### 2.6. Gut Microbiota Profiling

Bacterial profiling of cecal samples was carried out as described previously [32]. In short, nucleic acids were isolated from frozen cecal samples (*n* = 6 mice/group, equal number of mice per cage) with the MoBio PowerMag Microbiome kit (Carlsbad, CA, USA) and optimized for high-throughput processing. Samples were quantified via the Qubit Quant-iT dsDNA High Sensitivity Kit (Invitrogen; Life Technologies, Waltham, MA, USA) to ensure minimum concentration and DNA mass. Next, samples were PCR-amplified to enrich the 16S v4 rDNA region using 2 differently barcoded V4 fusion primers, and then pooled and sequenced on a Miseq^®^ instrument (Illumina, San Diego, CA, USA) for 250 cycles using custom primers for pair-end sequencing (Second Genome). Operational taxonomic units (OTU) were sequenced, pair-end reads were merged and compared to an in-house database using USEARCH with an identity of ≥99% required for each unique strain [33]. Non-strain sequences remaining were quality filtered and dereplicated using USEARCH. Unique sequences were then clustered at 97% similarity by UPARSE, and a representative consensus sequenced per de novo OTU was determined. Representative OTU sequences were assigned taxonomic classification via Mothurs Bayesian classifier trained against the Greengenes reference database of 16S rRNA gene sequences.

### 2.7. Statistical Analyses 

For all continuous variables, data were checked for the assumptions of normality and equal variance, transformed if needed, and then analyzed using SAS Version 9.4 (SAS Institute, Cary, NC, USA). To examine the ability of the crude polyphenol extract to counter the effects of estrogen deficiency in osteopenic ovariectomized mice and to compare the response to that of dried plum, a 1-way ANOVA was performed on data from the 5- and 10-week cohorts. When the *p*-value was significant (*p*-value < 0.05), post-hoc analyses using Fisher’s Test were performed. 

Additionally, among the OVX mice a 2 × 2 factorial was embedded within the experimental design with PP and CHO as factors. The objective of the factorial was to examine the simple effects of PP and CHO compared to the OVX-Con or the OVX-Crude extract treated mice in the 5- and 10-week cohorts. To accomplish this, least square means were calculated by using a proc mixed model procedure with a slice function to determine the simple effects of PP or CHO alone (vs. OVX-Con) or in combination (PP or CHO vs. crude extract). Data are presented as mean ± standard error (SE) unless indicated otherwise and α was set at 0.05.

For the microbiota data, Principal Coordinate Analysis (PCoA) was performed using sample-to-sample dissimilarity values calculated based on the Bray-Curtis method [34]. PERMANOVA was used to test microbial community differences between groups. When differences were detected, Kruskal–Wallis or Dunn’s post-hoc tests were used to determine which groups differ. The univariate differential abundance of OTUs at the phylum and family level was tested using a negative binomial noise model for the overdispersion and Poisson process intrinsic to the data, as implemented in the DESeq2 package [35] and previously described for microbiome applications [36]. DESeq2 was run using default settings, and *p*-values were corrected for false discovery rates using the Benjamini–Hochberg procedure [37]. OTUs with the adjusted *p*-value < 0.05 and more than one log 2-fold change were noted and the four most abundant phyla representing >99% of the population and eight most abundant families are reported here. 

## 3. Results

### 3.1. Dried Plum and Crude Extract Influence on Body Weight, Bone Density and Bone Biomarkers of Ovariectomized Mice

Two weeks post-surgery and prior to the initiation of treatment, OVX mice in both the 5 and 10 week cohorts exhibited the expected increase (~7%) in body weight compared to the ovary intact, sham-operated (Sham) mice (Table 1). After 5 weeks, the OVX-induced increase in weight was reduced in mice consuming diets supplemented with the DP (*p =* 0.0002) as well as the crude extract (*p =* 0.0428) compared to OVX-Con mice. Weight gain in response to OVX was attenuated to a greater extent in the DP treated group than the crude extract at this early time point. However, after 10 weeks, only the DP treated group had significantly lower body weight than OVX-Con mice (Table 1). Importantly, no differences were observed in food intake between treatment groups in either the 5- or 10-week cohorts (data not shown). Uterine weight was reduced in all OVX groups (*p* < 0.01), which was anticipated with estrogen deficiency (Table 1) and there were no effects of the DP or crude extract treatments on uterine weight.

Whole body BMD was decreased (*p* < 0.0001) in OVX mice consuming the Con diet in the 5 and 10 week cohorts compared to the Sham groups (Table 1). This effect on BMD conincided with a decrease in BMC in the 5 and 10 week groups (*p <* 0.001) and a decrease in BMA (*p =* 0.0005) in the 10 week cohort only (Table S1). At 5 weeks both the DP and crude extract groups exhibited greater BMD than the OVX mice on the Con diet, but only the crude extract diet restored BMD to the level of the Sham group (Table 1). By 10 weeks, both the DP and crude extract treatment improved whole body BMD compared to OVX mice on the Con diet and restored BMD to that of the Sham group. These improvements in BMD exhibited by the DP and the crude extract supplemented groups in both the 5 and 10 week cohorts resulted from significantly improved BMC and BMA compared to the OVX-Con groups (Table S1).

The observed improvements in bone density when OVX mice were fed diets supplemented with DP or crude extract coincided with alterations in serum biomarkers of bone formation and resorption (Table 1). P1NP was significantly suppressed in the OVX mice, regardless of whether the mice were consuming the control diet or diets supplemented with DP and crude extract at the end of 5 weeks of treatment. After 10 weeks, serum P1NP had normalized in th OVX mice consuming the the control and crude extract supplemented diets, and only the DP treated group exhibited a decrease in P1NP (*p* < 0.0001) compared to the Sham group (Table 1). In the 5 and 10 week cohorts, only DP suppressed (*p* < 0.01) TRAcP compared to the Sham and OVX-Con groups (Table 1). It should be noted that at this time point, serum TRAcP was not elevated in the OVX-Con compared to the Sham controls. The crude extract diets signficantly suppressed the OVX-induced increase in serum TRAcP after 10 weeks of treatment to a level that was similar to the Sham group. These findings indicate that at 5 weeks of treatment, OVX mice consuming DP supplemented diets exhibited a suppression in the activity of osteoblasts and osteoclasts compared to the Sham group. This suppressive effect of DP on osteoblast continued even after 10 weeks of treatment. In contrast, OVX mice consuming the diet supplemented with the crude extract of DP’s polyphenols normalized osteoclast and osteoblast activity after 10 weeks.

### 3.2. Dried Plum and Crude Extract Diets Improve Trabecular and Cortical Bone Microarchitecture of Ovariectomized Mice

Trabecular bone loss is one of the hallmarks of ovarian hormone deficiency. In the 5- and 10-week cohorts, trabecular BV/TV was reduced in distal femur metaphysis (*p* < 0.05) as well as in the lumbar vertebral body (*p* < 0.0001) of the OVX-Con compared to the Sham groups (Table 2). At both sites, this decrease in BV/TV coincided with significant decreases in TbN and TbTh and increases in TbSp after 5 weeks. Similar alterations in TbN and TbSp were evident in the femur as well as the lumbar vertebra after 10 weeks, but TbTh was decreased only in the vertebra (Table 2). These morphometric changes in the trabecular bone are consistent with those anticipated in OVX mice [19,23]. 

Incorporating DP into the diet prevented trabecular bone loss and, in some cases, reversed trabecular bone loss in OVX mice. Within the distal femur metaphysis, mice consuming the DP supplemented diet exhibited an increase (*p* < 0.01) in BV/TV and TbN after 5 weeks compared to the OVX-control group (Table 2). This effect of DP on trabecular bone resulted in a BV/TV that was similar to the Sham group, which is consistent with its ability to restore trabecular bone in osteopenic OVX animals as we have previously reported [16,38]. Furthermore, DP significantly increased TbN and decreased TbSp within this region of the bone to an extent greater than Sham mice. After 10 weeks of treatment, DP supplementation increased BV/TV in the femur metaphysis compared to the OVX-Con group and resulted in a 45% increase (*p* < 0.0001) in BV/TV above that of the Sham group (Table 2). These improvements coincided with an increase in TbN (*p* < 0.001) and decrease in TbSp (*p* < 0.001) compared to the OVX-Con mice as well as the Sham. Similarly, within the lumbar vertebra, DP not only significantly improved BV/TV compared to the OVX-Con mice, but also restored trabecular bone in the Sham 5 and 10 week cohorts (Table 2). These improvements in trabecular bone within the vertebra occurred in conjunction with increases (*p* < 0.01) in TbTh at both time points and improved TbN and TbSp (*p* < 0.01) at 10 weeks.

The crude extract mimicked the effects of DP on trabecular bone within the lumbar vertebra in the 5- and 10-week cohorts. Significant improvements in all four morphometric parameters, including BV/TV, TbTh, TbN, and TbSp were observed (Table 2). In the trabecular bone of the distal femur metaphysis, the crude extract had similar effects as DP after 5 weeks on BV/TV as well as TbTh, TbN, and TbSp. However, after 10 weeks of treatment, the crude extract increased BV/TV (*p* < 0.0001) within the distal femur above that of the OVX-Con and Sham groups, but not to the extent that was observed with DP (Table 2). Likewise, TbN and TbSp were significantly improved over the OVX-Con and were comparable to the Sham group, but the magnitude of the response with the crude extract was not as great as that of with the DP.

Cortical bone was evaluated at the femur mid-diaphysis. In the 5- and 10-week cohorts, only the cortical area (Table 2) was significantly reduced in response to OVX compared to the Sham-operated mice and no other indices of cortical bone were changed (Table S1). Interestingly, the crude extract outperformed the DP treatment on cortical bone parameters. After 5 weeks, the OVX mice consuming the diet supplemented with the crude extract exhibited an increase in cortical thickness compared to both the Con and DP supplemented groups and a trend for increasing (*p* = 0.0601) cortical area (Table 2). By 10 weeks the cortical area and thickness were significantly greater in the OVX mice consuming the diet supplemented with the crude extract compared to the OVX-DP group. Neither the medullary area nor the cortical bone porosity was affected by treatments at 5 weeks, but by 10 weeks DP tended to decrease the medullary area and the crude extract decreased cortical porosity compared to the OVX-Con mice (Table S1). 

### 3.3. PP and CHO Fractions Alter Body Weight, Bone Density, Biomarkers, and Trabecular and Cortical Bone Microarchitecture

**Figure 2:**
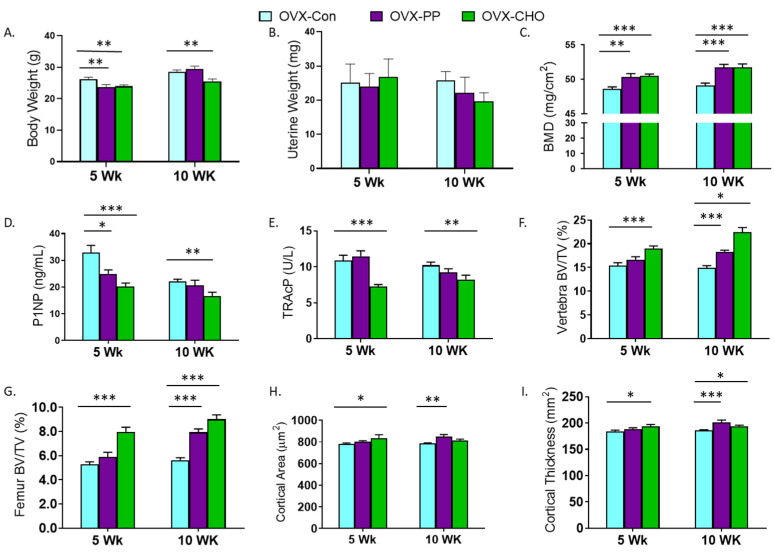
Simple effects of ovariectomized (OVX) mice fed diets supplemented with PP (OVX-PP) or CHO (OVX-CHO) compared to the control diet (OVX-Con) for 5 or 10 weeks on (**A**) body weight, (**B**) uterine weight, (**C**) whole body bone mineral density (BMD), serum (**D**) N-terminal propeptide of procollagen type 1 (P1NP) and (**E**) tartrate resistant acid phosphatase (TRAcP), trabecular bone volume (BV/TV) in the (**F**) lumbar vertebra and (**G**) distal femur metaphysis, and cortical (**H**) area and (**I**) thickness in the femur mid-diaphysis. Data are presented as mean ± SE (*n* = 10–12 mice/group except for microCT analyses of 8 mice/group). Lines indicating statistically significant differences between OVX-PP or OVX-CHO and the OVX-Con group in the 5 and 10 week cohorts are noted with * *p* < 0.05, ** p < 0.01, or *** p < 0.001.

A primary objective of this study was to determine how the PP and CHO fractions of the crude extract contribute to key bone structural and metabolic outcomes. First, we evaluated the simple effects of the PP and CHO on body and uterine weight, and bone metabolic and structural changes compared to the OVX-Con group. Both the PP and CHO fractions attenuated weight gain associated with OVX at 5 weeks, but this response was sustained at 10 weeks by the CHO only (Figure 2A). Uterine weight was not affected by the PP or the CHO in the 5- or 10-week cohorts (Figure 2B). Whole body 

BMD was increased (*p* < 0.001) in both cohorts by the PP and CHO fractions (Figure 2C). This increase in BMD coincided with a higher BMC (*p* < 0.05) at both time points, but BMA was also increased with the CHO fraction at 5 weeks (Table S2). Compared to the OVX-Con group, serum P1NP was suppressed with the PP (*p* < 0.05) and CHO (*p* < 0.001) fractions at 5 weeks, but only the CHO (*p* < 0.01) treatment after 10 weeks (Figure 2D). TRAcP was also significantly depressed with CHO at both 5 and 10 weeks, and the PP fraction had no effect (Figure 2E). 

Evaluation of trabecular bone in the lumbar spine (Figure 2F) and distal femur metaphysis (Figure 2G) revealed an early increase in BV/TV at 5 weeks with the CHO treatment, but by 10 weeks improvements in BV/TV had occurred at both sites in response to the PP and CHO fractions. In the distal femur metaphysis, a significant increase in TbN and decrease in TbSp, with no change in TbTh, occurred with the CHO fraction at both time points (Table S2). Within the spine, TbTh was the only parameter increased with CHO in the 5 week cohort, but by 10 weeks significant increases in TbTh and TbN and a decrease in TbSp were exhibited. PP treatment increased TbTh within the lumbar spine after 5 and 10 weeks, but no other statistically significant changes were noted. Likewise, in the distal femur PP did not alter TbTh, TbN, or TbSp (Table S2). 

Cortical bone was assessed in the femur mid-diaphysis. Increases in cortical area were exhibited in the 5 week cohort with CHO and in the 10 week cohort with PP (Figure 2H). Similar responses were observed in cortical thickness with the PP fraction, but the mice consuming the CHO fraction had greater cortical thickness in both the 5- and 10-week cohorts (Figure 2I). The area of the medullary cavity was not affected by treatments; however, cortical bone porosity was reduced (*p* = 0.0068) after 10 weeks of treatment with the PP fraction (Table S2).

### 3.4. Comparison of PP and CHO Effects to the Crude Extract on Bone Structural and Metabolic Outcomes

**Figure 3:**
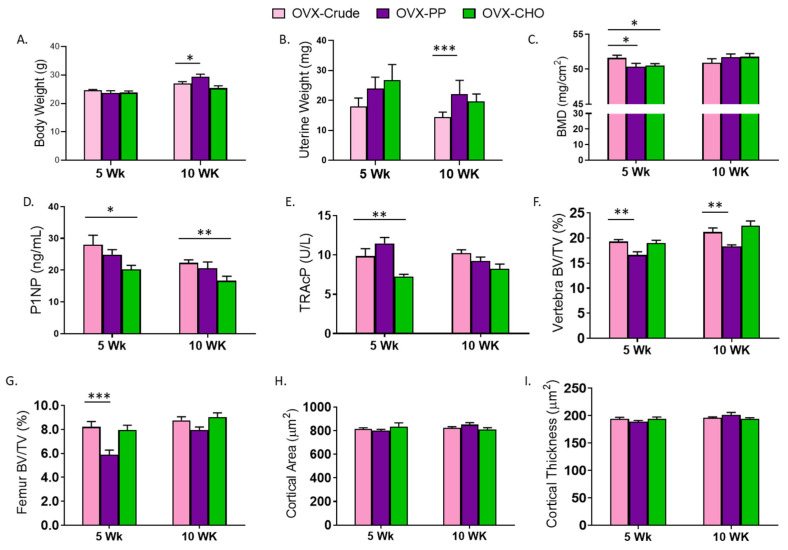
Simple effects of PP (OVX-PP) and CHO (OVX-CHO) compared to the crude extract diet (OVX-Crude) on (**A**) body weight, (**B**) uterine weight, (**C**) whole body bone mineral density (BMD), (**D**) serum N-terminal propeptide of procollagen type 1 (P1NP), (**E**) tartrate resistant acid phosphatase (TRAcP), trabecular bone volume (BV/TV) in the (**F**) lumbar vertebra and (**G**) distal femur metaphysis, and (**H**) cortical area and (**I**) cortical thickness in the femur mid-diaphysis in OVX mice fed diets for 5 or 10 weeks. Data are presented as means ± SE (*n* = 10–12 mice/group except for microCT analyses of 8 mice/group). Lines indicating statistically significant differences between OVX-PP or OVX-CHO and the OVX-Con group in the 5 and 10 week cohorts are noted with * *p* < 0.05, ** *p* < 0.01, or *** *p* < 0.001.

To further understand the contributions of the PP and CHO fractions within the crude extract, the simple effects of the fractions were compared to the extract. There were no differences in body weight between mice consuming the diet with the CHO and the crude extract in the 5- and 10-week cohorts suggesting that the ability of the crude extract to attenuate weight gain with OVX is attributed in part to the CHO (Figure 3A). In contrast, a signicant increase in body weight was exhibited in the group receiving the PP supplemented diet compared to the OVX-crude treated group for 10 weeks. Although no statistically significant effects of either fraction were noted previously in comparison to the OVX-Con cohorts, uterine weight was greater in the PP compared to the crude extract group at 10 weeks (Figure 3B). This appears to result from the numerical decrease in uterine weight associated with the crude exact (Table 1) as opposed to an estrogenic effect of the PP. Interestingly, neither the PP nor CHO fraction performed as well as the combination in the crude extract in restoring whole body BMD at 5 weeks, but by 10 weeks there were no differences between the groups (Figure 3C). As indicated by the serum bone biomarkers, the PP fraction mimicked the effects of the crude extract on P1NP (Figure 3D) and TRAcP (Figure 3E). However, mice receiving the diets supplemented with CHO alone exhibited a reduction in serum P1NP (*p* = 0.0141) and TRAcP (*p* = 0.0094) at 5 weeks, and the P1NP response to the CHO persisted at 10 weeks of treatment. 

Next the contributions of the PP and CHO fractions on the trabecular and cortical bone phenotype were assessed. Within the lumbar vertebra, the CHO fraction had similar effects as the crude extract on trabecular BV/TV in the 5- and 10-week cohorts (Figure 3F). The PP fraction alone was unable to restore BV/TV (*p* < 0.01) to that of the mice receiving the crude extract at this site. No differences in trabecular bone parameters of the lumbar vertebra including, TbTh, TbN, and TbSp were detected between the PP and CHO fractions and the crude extract in either the 5- or 10-week cohort (Table S2). The treatment effects of the PP and CHO fractions on the distal femur were analogous to those observed in the lumbar vertebra at 5 weeks, but by 10 weeks both fractions had comparable effects to the crude extract (Figure 3G). In the distal femur, the CHO fraction performed similarly to that observed in the vertebra, but the PP fraction failed to restore TbN (*p* = 0.001) or decrease TbSp to the extent that was observed with the crude extract in either cohort. 

Both fractions contributed to the effects of the crude extract on cortical bone. There were no differences in cortical bone area (Figure 3H), thickness (Figure 3I), or medullary area (Table S2) of the mice receiving the diets supplemented with the fractions compared to those receiving the crude extract. Cortical porosity was however, increased (*p* = 0.0229) in the 5 week cohort treated with the PP fraction, but not after 10 weeks. Cortical porosity was not altered after 5 or 10-weeks of treatment with the CHO fraction indicating this fraction mirrored the response observed with the combination of CHO and PP in the crude extract (Table S2).

### 3.5. Gut Microbiota and Dietary Supplementation with Dried Plum and Its Bioactive Components

**Figure 4:**
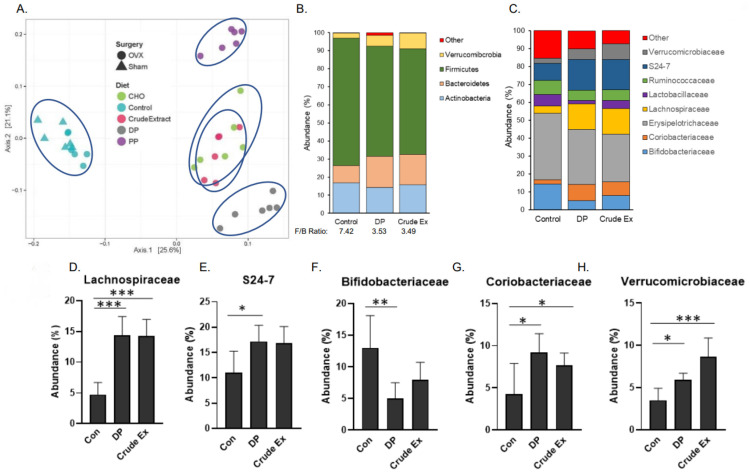
Cecal microbiota β-diversity based on Bray–Curtis distance of operational taxonomic units using (**A**) principal coordinate (weighted ordination) analysis (PCoA) of the five diet groups with the two groups on control diet denoted by Sham-Control Δ and OVX-Control O. The composition of most abundant taxa at the (**B**) phylum level with the Firmicutes to Bacteroidetes (F/B) ratio and the (**C**) family level comparing OVX mice consuming diets supplemented with dried plum (DP) or crude extract (Crude Ex) to OVX mice consuming the Control (Con) diet. The relative abundance of (**D**) *Lachnospiraceae*, (**E**) *S24-7*, (**F**) *Bidifidobacteriaceae*, (**G**) *Coriobacteriaceae*, and (**H**) *Verrucomicrobiaceae* are shown. Differences between groups (*n* = 6 mice/group) are indicated with asterisks when the Kruskal–Wallis test and Dunn’s post-hoc tests were significant (* *p* < 0.05, ** *p* < 0.01, *** *p* < 0.001).

Analysis of the cecal microbiota were performed on the 5 week cohort. Using the Bray–Curtis distance metrics, the β-diversity of the cecal microbiota of all groups was assessed. As shown, mice consuming the control, dried plum and crude extract diets clustered into three distinct groups with the mice on the crude extract and DP appearing to be more similar to each other than to the control (Figure 4A). Among the four diet groups in the 2 × 2 embedded factorial, mice consuming the PP fraction diet also formed a distinct cluster from the control and crude extract groups. However, mice consuming the diets supplemented with the crude extract and the CHO fraction did not separate, suggesting that the effects of the crude extract on the microbiota are primarily affected by the CHO component of the extract (Figure 4A).

We next explored alterations in the cecal microbiota in response to the DP and crude extract supplemented diets compared to the OVX-Con diet. The weights of the cecal contents were significantly increased by dried plum (90%) and the crude extract (40%) compared to mice on the control diet (Table 1). PERMANOVA indicated that DP and crude extract contributed to β-diversity (*p* < 0.001) compared to the control diet. It should be noted that PERMANOVA also revealed that despite being on the control diet, surgery status (i.e, OVX vs. Sham) contributed significantly (*p* < 0.001) to β-diversity (data not shown). The most abundant phyla are shown in Figure 4B and the Firmicutes to Bacteroidetes was ratio reduced from 7.42 in the OVX mice receiving the control diet to 3.53 and 3.49 in the mice consuming the diets supplemented with DP and crude extract, respectively. 

Next the alterations in taxa at the family level induced with DP and crude extract, focusing on the 8 most abundant families, were investigated (Figure 4C). Even though the abundance of taxa in the Firmicutes phyla overall was reduced in the mice consuming the DP and crude extract, the abundance of the *Lachnospiraceae* family, which producethe SCFA butryate, was increased (*p* < 0.001) in the mice consuming the DP and crude extracts (Figure 4D). Among members of the Bacteroidetes family, *S24-7* increased in abundance (*p* < 0.05) in the mice consuming DP, but the increase did not reach the level of statistical significance (*p* = 0.11) in the mice treated with the crude extract (Figure 4E). *Bifidobacteriaceae*, however, were less abundant (*p* < 0.05) in the mice consuming the DP compared to the control group (Figure 4F). Other significant shifts in the cecal microbiota associated with the DP and crude extract treatments included an increase in *Coriobacteriaceae* (Figure 4G) and *Verrucmibrobiaeae* (Figure 4H). Within these eight most abundant families, no statistically significant changes in the *Ruminococcaceae*, *Lactobacillaceae* or *Erysipelotrichaceae* were noted (data not shown).

**Figure 5:**
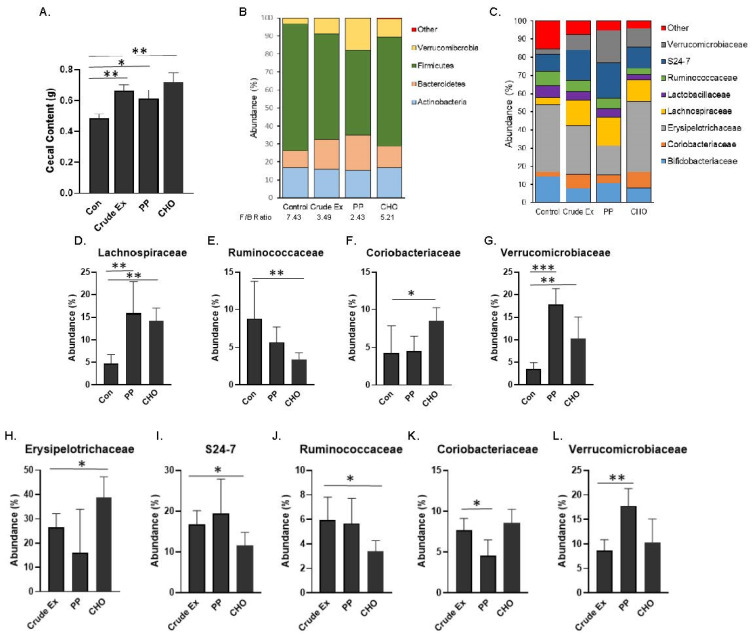
Following 5 weeks, simple effects of diets supplemented with the polyphenol (PP) fraction and carbohydrate (CHO) fraction compared to the crude extract or control (Con) diets on the (**A**) cecal content weights. Composition of most abundant taxa at the (**B**) phylum level including the Firmicutes to Bacteroidetes (F/B) ratio and the (**C**) family level. The relative abundance of (**D**) *Lachnospiraceae* (**E**) *Ruminococcaceae*, (**F**) *Coriobacteriaceae*, and (**G**) *Verrucomicrobiaceae* taxa of the PP or CHO treated mice were first compared to the Con diet. Next, the relative abundance of (**H**) *Erysipelotrichaceae*, (**I**) *S24-7*, (**J**) *Ruminococcaceae*, (**K**) *Coriobacteriaceae*, and (**L**) *Verrucomicrobiaceae* taxa of the PP or CHO treated mice were compared to the mice on the Crude Ex. Differences between groups (*n* = 6 mice/group) are indicated with asterisks when the Kruskal–Wallis test followed by Dunn’s post-hoc tests were significant (* *p* < 0.05, ** *p* < 0.01, or *** *p* < 0.001).

Within the OVX groups that make up the embedded 2 × 2 factorial, the effects of dietary treatments on the gut microbiota were investigated. Cecal content weights were significantly increased with the crude extract, PP and CHO compared to the control groups and no differences were noted between treatments (Figure 5A). The most abundant phyla, which make up >99% of the murine microbiota, are shown (Figure 5B). The Firmicutes to Bacteroidetes ratio was decreased in mice consuming diets supplemented with the crude extract (3.49), PP fraction (2.43) or CHO fraction (5.21) compared to the mice consuming the control diet (7.43). 

To understand the effects of the PP and CHO fractions on the cecal microbiota taxa, mice consuming the diets supplemented with the fractions were first compared to the mice on the control diet. The changes in the most abundant families are shown in Figure 5C. Within the Firmicutes, *Lachnospiraceae* was increased (*p* < 0.01) by both the PP and CHO fractions (Figure 5D). *Ruminococcaceae* taxa of the Bacteroidetes phylum was reduced (*p* < 0.05) with the CHO only (Figure 5E). *Coriobacteriaceae* and *Verrucomicrobiaceae* were significantly increased in the mice consuming the diet supplemented with the CHO fraction compared to the Con diet (Figure 5F–G). Although the PP supplemented diet did not significantly alter taxa within the *Coriobacteriaceae* family, the *Verrucomicrobiaceae* family was 6× greater in the mice consuming the PP diet compared to the control diet. There were no significant effects of either fractions on the *Bifidobacteriaceae*, *Lactobacillaceae*, *S24-7*, or *Erysipelotrichaceae* families after 5 weeks (data not shown). 

Shifts in the most abundant families induced with the PP and CHO fractions were next compared to mice fed the diet supplemented with the crude extract. The abundance of *Erysipelotrichaceae* was increased whereas the abundance of *S24-7* and *Ruminococcaceae* was reduced (*p* < 0.05) in the mice fed the CHO fraction; however, these shifts did not occur in mice receiving the PP fraction (Figure 5H–J). The PP supplemented diet did induce a reduction (*p* < 0.05) in the abundance of *Coriobacteriaceae* (Figure 5K) and an increase (*p* < 0.01) in the abundance of *Verrucmicrobiaceae* (Figure 5L) in comparison to the mice receiving the crude extract. Together these findings suggest that the PP and CHO within the crude extract differentially contribute to the alterations in the microbial taxa.

### 3.6. Short Chain Fatty Acids Production Induced by Dried Plum and Its Bioactive Components

In the 10-week cohort, cecal SCFAs analyses showed that dried plum supplementation induced an ~10-fold increase in total SCFAs compared to the Sham and OVX mice on the control diet (Table 3). Acetate, the most abundant SCFAs in the cecum was increased by dried plum compared to the OVX-Con group, as were propionate, n-butyrate, and i- and n-valerate (Table 3). Notably i-butyrate was not. The crude extract increased all of the SCFAs that were assessed. The magnitude of the increase in acetate, propionate, n-butyrate, and n-valerate was greater in the DP treated mice compared to those receiving the crude extract. Interestingly, the crude extract diet seemed to have a more pronounced effect on the i-isoforms of butyrate and valerate, whereas the n-isoforms were increased to a greater extent with the DP diet. 

**Figure 6:**
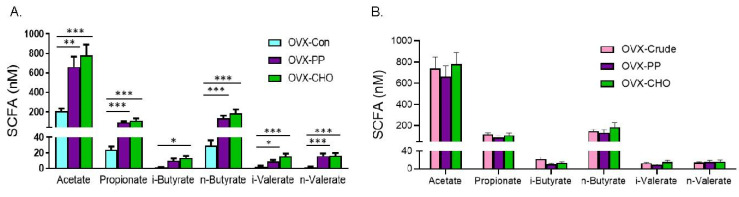
Simple effects of PP and CHO treatment on cecal short chain fatty acids of OVX mice following 10 weeks of treatment compared to OVX mice on (**A**) control (Con) diet and diet supplemented with the (**B**) crude extract (Crude). Data are presented as mean ± SE (*n* = 10 mice/group). Statistically significant differences are noted by * *p* < 0.05, ** *p* < 0.01, or *** *p* < 0.001.

In order to examine the simple effect of the PP and CHO fractions on cecal SCFA, comparisons were first made with the mice consuming the control diet. Total SCFA were increased by the PP (918.93 ± 149.12 nM) and the CHO (1120.67 ± 172.92 nM) fractions compared to OVX-Con (264.92 ± 38.68 nM). Likewise, the PP and CHO fractions increased acetate, propionate, n-butryate, i- and n-valerate compared to the OVX-Con mice (Figure 6A). Only the mice on the PP supplemented diet failed to exhibit a significant increase in i-butyrate. Compared to the mice on the diet supplemented with the crude extract (Figure 6B), there were no significant differences in the total SCFA (PP = 918.93 ± 149.12 nM or CHO = 1120.67 ± 172.92 nM vs. crude extract = 1054.95 ± 115.04 nM) or any of the individual SCFAs that were examined. Together these findings reveal that the PP as well as the CHO contribute to the increase in SCFAs observed with the crude extract treatment, but the effects do not appear to be additive. 

## 4. Discussion

In this study, we investigated how the carbohydrates and polyphenolic compounds in dried plum mitigate the negative effects of ovarian hormone deficiency on bone and their prebiotic activity. We first confirmed that our prior observation [27] demonstrating the efficacy of the crude extract in the OVX rat model, held true in the OVX mouse. The dried plum and crude extract produced very similar whole BMD and trabecular bone effects across both time points. The only exception being that the trabecular bone response was greater with the dried plum in the femur following 10 weeks of treatment, and yet the dried plum and crude extract elicited increases in trabecular bone that were 31% and 25% greater, respectively, than the Sham-operated animals. While this could be interpreted as anabolic, the time course reveals a decline in the Sham cohort’s trabecular bone occurred between 5 and 10 weeks, which is consistent with the onset of trabecular bone loss in female C57BL/6 mice [39]. Interestingly, the crude extract had a greater effect than dried plum on indices of cortical bone at both time points. The bone structural changes coincided with reduced serum bone formation and resorption biomarkers with dried plum, a response consistent with most of the clinical and pre-clinical studies of postmenopausal osteoporosis [8,10,16]. In contrast, the crude extract produced a more modest reduction in bone resorption at both timepoints, and bone formation had normalized in the 10-week cohort. This may account for the differences in cortical changes that were observed. Bone histomorphometry, considered the gold standard for evaluating the bone metabolic response to treatment, has shown that dried plum decreases osteoclast and osteoblast number, and suppresses bone formation, which is consistent with these findings [40]. We are not aware of reports using dynamic bone histomorphometric techniques to determine the bone cellular response to the crude extract. 

After characterizing the bone structural and metabolic changes occurring in response to the crude extract compared to the whole fruit, our focus turned to studying how the PP and CHO fractions of this extract contribute to the bone outcomes. Previously, our laboratory and others have attributed the bone promoting properties of the fruit primarily to its polyphenols [17,20,21,27]. In this study, both fractions improved BMD at each timepoint, but a longer duration was required for the individual fractions to achieve the same response as the crude extract. Trabecular bone was increased with the CHO early and by both fractions by the later time point. The cortical bone response in the appendicular skeleton followed the same pattern as that of trabecular bone. The bone remodeling cycle of the mouse is approximately two weeks in duration, which may indicate the mechanisms through which the PP manifests into bone structural changes requires more remodeling cycles than the CHO. Interestingly, the CHO fraction had a greater effect on bone resorption than the crude extract and suppressed P1NP throughout the study. The suppressive effects of the PP fraction on P1NP were temporal and allowed bone formation to normalize. This type of biphasic response on osteoblast activity has been previously reported with dried plum based on bone histomorphometry [40]. The absence of a systemic anti-resorptive effect of the PP fraction differs from our previous in vivo and in vitro reports, and those of others demonstrating that the polyphenolic compounds in dried plum down-regulate osteoclastogenesis by inhibiting NFATc1 [16,17,41]. Furthermore, despite the effectiveness of both fractions of the extract, it was evident that the effects of the CHO and PP were not additive in the current study. 

Due to reports that the parent polyphenolic compounds are not well absorbed and the effectiveness of the CHO fraction on bone observed in this study, the prebiotic potential of dried plum and its bioactive components were explored. The cecal microbiota analyses, performed at the earlier timepoint, revealed that the five different diets used in this study clustered separately, with the exception of the crude extract and CHO. It is noteworthy that the bone phenotype associated with feeding the CHO fraction consistently aligned with the skeletal response observed with the crude extract at this time point. Although they did not cluster together in the principal coordinate analysis, the response to the DP and crude extract were comparable in terms of the alterations induced in the most abundant families. Both the DP and crude extract increased the abundance of *Lachnospiraceae*, *Coriobacterieaceae*, and *Verrucomicrobiaceae*. Members of the *Lachnospiraceae* family are anaerobic fermenters and produce butyrate as well as other SCFAs [42]. Other families that were enhanced included *Coriobacterieaceae,* that are reported to play an important role in the bile salts, steroids, and polyphenols metabolism [43,44], and *Verrucomicrobiaceae* that produce propionate by way of the succinate pathway [45]. Also worth noting, the abundance of *S24-7* was increased and *Bifidobacterieaceae* was decreased with DP, with similar trends produced by the extract. *S24-7*, also known as *Muribaculaceae*, is recognized as one of the most abundant families in the mouse GI tract and is highly involved in complex carbohydrate degradation [46]. Thus, the more modest effect of the crude extract on *S24-7* may be attributed to the extract being matched to the polyphenolic content of the dried plum and not individual carbohydrates. The decrease in *Bifidobacterieaceae* observed in the current study is not in line with the report of Lever and colleagues [47] showing an increase in *Bifidobacterieaceae* in healthy adults consuming dried plums (80 or 120 g/d) and no change in SCFAs. Both fractions contributed to the microbiota response and replicated most of the same shifts induced with the dried plum and crude extract. The exception to this is the increase in *Erysipelotrichaceae* and decrease in *Ruminococcaceae* that occurred with the CHO treatment. *Erysipelotrichaceae* is considered immunogenic, but there is debate as to its role due perhaps in part to the fatty acid biosynthesis by some of its taxa [48]. In humans, members of the *Ruminococcaceae* family have the ability to degrade cellulose and produce SCFA, and has been associated with improved energy metabolism in mice [49]. Interestingly, while neither of these shifts in *Erysipelotrichaceae* or *Ruminococcaceae* are considered beneficial, when CHO and PP were combined in the crude extract or the fruit, neither of these populations were affected.

In addition to altering the microbiota, the individual fractions also increased cecal SCFA at 10 weeks. With the exception of i-butyrate, both fractions increased the SCFAs that were assessed and there were no differences between the individual fractions and the crude extract. Although the experiments in the current study were not designed to discern whether SCFAs were the driving mechanism through which bone was protected in ovarian hormone deficiency, the alterations in SCFA were consistent with the observed bone changes. This is further supported by the fact that at the early time point, it was primarily the CHO fraction that enhanced bone microarchitecture. We analyzed the remaining cecal samples (*n* = 3–5/group) that had not been allocated for microbiota profiling at 5 weeks and found that only the CHO fraction significantly increased SCFAs (i.e., acetate, propionate, and n-butyrate) at this earlier time point (data not shown). When comparing acetate, propionate, and n-butyrate, Lucas et al. [50] reported that n-butyrate and propionate were most effective at preventing bone loss in the OVX model by suppressing TRAF6 and NFATc1 that are essential in osteoclastogenesis. A large proportion of bone-related research has focused on butyrate. In addition to its anti-resorptive activity, butyrate also stimulated Wnt10b expression and thus, bone formation, by enlarging the T regulatory pool in the bone marrow [29]. Administration of butyrate has direct and indirect effects on bone cells. SCFAs have well-known benefits on the epithelium of the gastrointestinal tract and can mitigate the compromise in gut barrier integrity associated with estrogen deficiency [51]. Still, further mechanistic studies are warranted to determine the role of SCFAs in mediating the effects of dried plums and their bioactive components.

In summary, the findings of this study indicate that both the carbohydrate and polyphenol components of dried plum likely contribute to the fruit’s efficacy in preventing bone loss in a mouse model of postmenopausal osteoporosis. Although the polyphenolic compounds appear to play an important role, it is evident that the carbohydrate-containing fraction may also contribute to the fruit’s ability to counter the effects of estrogen deficiency on bone. Based on the results of this work, it is evident that the polyphenols as well as the carbohydrates convey prebiotic activity in terms of their beneficial effects on bone, the gut microbiota and the production of SCFAs. Characterization of the carbohydrate components of dried plum has been previously reported [52], but may warrant revisiting in the context of prebiotics and bone health. The increase in SCFAs induced with both components suggests potential benefits on mineral absorption, immune modulation, as well as enhanced gut barrier integrity due to these components, which could be explored in future animal as well as human studies. It is worth noting that this study does not eliminate the possibility that other gut-derived metabolites, aside from SCFAs, are responsible for the bone phenotype reported here. Furthermore, other nutrient or non-nutrient components of the fruit contained within the fractions could be contributing to the effects, despite our best efforts to control for these factors. 

## Figures and Tables

**Table 1:** Body weight, bone density, and bone biochemical markers following 5 or 10 weeks of dietary supplemention with dried plum or the crude extract.

	Sham-Con	OVX-Con	OVX-DP	OVX-Crude	*p*-Value
**5 Weeks**					
Body weight					
Pre-treatment (g)	20.80 ± 0.27 ^b^	22.30 ± 0.29 ^a^	21.75 ± 0.34 ^a^	22.25 ± 0.20 ^a^	0.0011
Final (g)	21.52 ± 0.30 ^d^	26.18 ± 0.65 ^a^	23.13 ± 0.73 ^c^	24.65 ± 0.29 ^b^	<0.0001
Uterine weight (mg)	112.12 ± 3.73 ^a^	24.38 ± 5.10 ^b^	26.54 ± 5.72 ^b^	21.93 ± 4.70 ^b^	0.0016
Whole body BMD (mg/cm^2^)	51.65 ± 0.46 ^a^	48.62 ± 0.29 ^c^	50.55 ± 0.34 ^b^	51.61 ± 0.38 ^a^	<0.0001
Bone biomarkers					
P1NP (ng/mL)	56.91 ± 7.80 ^a^	32.97 ± 2.62 ^b^	26.87 ± 1.70 ^b^	27.97 ± 3.06 ^b^	<0.0001
TRAcP (U/L)	11.28 ± 1.01 ^a^	10.89 ± 0.72 ^a^	7.72 ± 0.39 ^b^	9.85 ± 0.96 ^ab^	0.0119
Cecal content weight (g)	0.48 ± 0.04 ^c^	0.48 ± 0.03 ^c^	0.91 ± 0.07 ^a^	0.67 ± 0.05 ^b^	<0.0001
**10 Weeks**					
Body weight					
Pre-treatment (g)	20.15 ± 0.23 ^b^	21.76 ± 0.19 ^a^	21.71 ± 0.39 ^a^	22.07 ± 0.25 ^a^	<0.0001
Final (g)	21.93 ± 0.40 ^c^	28.50 ± 0.63 ^a^	24.40 ± 0.62 ^b^	27.02 ± 0.68 ^a^	<0.0001
Uterine weight (mg)	80.86 ± 5.94 ^a^	31.86 ± 6.54 ^b^	28.85 ± 6.30 ^b^	14.43 ± 1.66 ^b^	<0.0001
Whole body BMD (mg/cm^2^)	52.00 ± 0.29 ^a^	49.09 ± 0.37 ^b^	51.16 ± 0.53 ^a^	50.92 ± 0.57 ^a^	0.0001
Bone biomarkers					
P1NP (ng/mL)	27.67 ± 2.27 ^a^	22.15 ± 0.77 ^ab^	17.73 ± 1.38 ^b^	22.32 ± 0.94 ^ab^	0.0003
TRAcP (U/L)	8.09 ± 0.21 ^b^	10.22 ± 0.44 ^a^	7.03 ± 0.33 ^c^	7.96 ± 0.44 ^bc^	<0.0001
Cecal content weight (g)	0.53 ± 0.04 ^c^	0.42 ± 0.04 ^c^	1.08 ± 0.05 ^a^	0.73 ± 0.04 ^b^	<0.0001

Data are presented as mean ± SE (*n* = 10–12 mice/group). *p*-values shown are based on 1-way ANOVA. Within a given row, groups that do not share the same superscript letter are significantly different from each other based on post-hoc analyses. Abbreviations: sham-operated (Sham), ovariectomized (OVX), control diet (Con), control diet supplemented with dried plum (DP), crude polyphenol extract (Crude), bone mineral density (BMD), precollagen type 1 N-terminal propeptide (P1NP), tartrate-resistant acid phosphate (TRAcP).

**Table 2:** Trabecular and cortical bone microarchitecture of the distal femur metaphysis, lumbar vertebra, and femur mid-diaphysis in mice fed diets supplemented with dried plum or the crude extract for 5 or 10 weeks.

	Sham-Con	OVX-Con	OVX-DP	OVX-Crude	*p*-Value
**5 weeks**					
Distal femur metaphysis					
BV/TV (%)	8.49 ± 0.74 ^a^	5.28 ± 0.21 ^b^	8.30 ± 0.83 ^a^	8.21 ± 0.45 ^a^	0.0044
TbTh (mm)	0.048 ± 0.001 ^a^	0.041 ± 0.001 ^b^	0.039 ± 0.001 ^b^	0.041 ± 0.001 ^b^	<0.0001
TbN (1/mm^2^)	3.43 ± 0.07 ^b^	3.09 ± 0.10 ^c^	3.83 ± 0.13 ^a^	3.81 ± 0.04 ^a^	<0.0001
TbSp (mm)	0.29 ± 0.01 ^b^	0.32 ± 0.01 ^a^	0.26 ± 0.01 ^c^	0.26 ± 0.003 ^c^	<0.0001
Lumbar vertebra					
BV/TV (%)	20.31 ± 0.69 ^a^	15.38 ± 0.64 ^b^	20.54 ± 0.41 ^a^	19.31 ± 0.39 ^a^	<0.0001
TbTh (mm)	0.048 ± 0.001 ^a^	0.043 ± 0.001 ^b^	0.049 ± 0.001 ^a^	0.048 ± 0.0004 ^a^	<0.0001
TbN (1/mm^2^)	4.41 ± 0.08 ^a^	3.94 ± 0.09 ^b^	4.10 ± 0.05 ^b^	3.95 ± 0.06 ^b^	0.0034
TbSp (mm)	0.228 ± 0.005 ^b^	0.260 ± 0.01 ^a^	0.245 ± 0.003 ^a^	0.255 ± 0.004 ^a^	0.0002
Femur mid-diaphysis					
Cortical area (μm^2^)	813.69 ± 15.66	780.03 ± 10.22	775.52 ± 14.54	815.34 ± 10.25	0.0601
Cortical thickness (μm)	193.11 ± 2.55 ^a^	184.44 ± 1.94 ^b^	186.00 ± 1.35 ^b^	193.90 ± 2.71 ^a^	0.0093
**10 weeks**					
Distal femur metaphysis					
BV/TV (%)	6.99 ± 0.37 ^c^	5.63 ± 0.20 ^d^	10.17 ± 0.58 ^a^	8.76 ± 0.30 ^b^	<0.0001
TbTh (mm)	0.044 ± 0.001	0.045 ± 0.001	0.043 ± 0.001	0.045 ± 0.001	0.6237
TbN (1/mm^2^)	3.94 ± 0.13 ^b^	2.81 ± 0.08 ^c^	3.66 ± 0.14 ^a^	3.28 ± 0.08 ^b^	<0.0001
TbSp (mm)	0.32 ± 0.01 ^b^	0.36 ± 0.01 ^a^	0.27 ± 0.01 ^c^	0.30 ± 0.01 ^b^	<0.0001
Lumbar vertebra					
BV/TV (%)	21.96 ± 0.95 ^a^	14.98 ± 0.43 ^b^	22.18 ± 0.95 ^a^	21.19 ± 0.84^a^	<0.0001
TbTh (mm)	0.053 ± 0.001 ^a^	0.047 ±0.0004 ^c^	0.050 ± 0.001 ^ab^	0.050 ± 0.001 ^bc^	0.0010
TbN (1/mm^2^)	3.94 ± 0.13 ^a^	3.37 ± 0.08 ^b^	4.00 ± 0.13 ^a^	3.88 ± 0.19 ^a^	0.0244
TbSp (mm)	0.26 ± 0.01 ^b^	0.30 ± 0.01 ^a^	0.25 ± 0.01 ^b^	0.26 ± 0.01 ^b^	0.0284
Femur mid-diaphysis					
Cortical area (um^2^)	823.59 ± 7.53 ^a^	785.12 ± 7.28 ^ab^	745.48 ± 48.03 ^b^	825.00 ± 9.80 ^a^	0.0390
Cortical thickness (um)	196.88 ± 1.79 ^a^	186.00 ± 1.18 ^ab^	174.40 ± 12.20 ^b^	196.00 ± 1.6 ^a^	0.0162

Data are presented as mean ± SE (*n* = 8 mice/group). *p*-values shown are based on 1-way ANOVA. Within a given row, groups that do not share the same superscript letter are significantly different from each other based on post hoc analyses. Abbreviations: bone volume (BV/TV), trabecular thickness (TbTh), trabecular number (TbN), trabecular separation (TbSp). Sham-operated (SHAM), ovariectomized (OVX), control diet (Con), diet supplemented with dried plum (DP), diet supplemented with crude polyphenol extract (Crude).

**Table 3:** Cecal Short Chain Fatty Acids in Mice Consuming the Diets Supplemented with Dried Plum or Crude Extract for 10 Weeks.

	Sham-Con	OVX-Con	OVX-DP	OVX-Crude	*p* Value
Total SCFA (nM)	341.15 ± 73.05 ^c^	264.92 ± 38.68 ^c^	3092.90 ± 550.92 ^a^	1054.95 ± 115.04 ^b^	<0.0001
Acetate (nM)	270.92 ± 56.99 ^c^	206.96 ± 27.08 ^c^	2259.18 ± 437.21 ^a^	739.25 ± 107.79 ^b^	<0.0001
Propionate (nM)	29.86 ± 6.87 ^c^	24.08 ± 4.19 ^c^	374.30 ± 64.38 ^a^	119.16 ± 13.27 ^b^	<0.0001
i-butyrate (nM)	1.98 ± 1.30 ^b^	1.01 ± 0.69 ^b^	6.61 ± 3.27 ^b^	21.69 ± 5.49 ^a^	0.0090
n-butyrate (nM)	31.76 ± 6.62 ^c^	29.25 ± 6.90 ^c^	421.53 ± 70.79 ^a^	147.40 ± 17.15 ^b^	<0.0001
i-valerate (nM)	4.65 ± 1.45 ^bc^	2.12 ± 1.27 ^c^	9.10 ± 2.95 ^ab^	12.80 ± 1.92 ^a^	0.0109
n-valerate (nM)	1.98 ± 1.30 ^c^	1.49 ± 0.75 ^c^	22.18 ± 4.01 ^a^	14.65 ± 1.64 ^b^	<0.0001

Data were analyzed using 1-way ANOVA and are presented as mean ± SE (*n* = 10 mice/group). Within a given row, groups that do not share the same superscript letter are significantly different from each other based on post hoc testing. Sham-operated (SHAM), ovariectomized (OVX), control diet (Con), diet supplemented with dried plum (DP), diet supplemented with crude polyphenol extract (Crude).

## Data Availability

Data associated with this manuscript will be made available upon request.

## Supplementary Materials

The following are available online at https://www.mdpi.com/article/10.3390/nu14091685/s1, Table S1. Additional DXA and MicroCT parameters of the trabecular and cortical bone in ovariectomized mice supplemented with dried plum or the crude extract for 5 or 10 weeks. Table S2: Simple effects of the PP and CHO components of the crude extract on other whole body DXA and trabecular and cortical microCT parameters.
