# Supplementary material for: Dried Plum’s Polyphenolic Compounds and Carbohydrates Contribute to Its Osteoprotective Effects and Exhibit Prebiotic Activity in Estrogen Deficient C57BL/6 Mice
Source: Nutrients. 2022 Apr 19;14(9):1685. doi: 10.3390/nu14091685 (PMC9102795; doi:10.3390/nu14091685)
Supplement: Supplementary file 1 [file nutrients-14-01685-s001.zip › nutrients-1667416-supplementary.pdf]

Table S1: Additional DXA and MicroCT parameters of the trabecular and cortical bone in ovariectomized mice supplemented with dried plum or the crude extract for 5 or 10 weeks.

	Sham-Con	OVX-Con	OVX-DP	OVX-Crude	P-Value
**5 weeks**					
Whole Body					
BMC (mg)	524.77 ± 11.34[b]	472.46 ± 7.47[c]	552.85 ± 9.09[ab]	567.27 ± 11.41[a]	**<0.0001**
BMA (cm^2)	10.17 ± 0.23[b]	9.72 ± 0.15[b]	10.94 ± 0.16[a]	10.99 ± 0.19[a]	**<0.0001**
Femur Mid-Diaphysis					
Medullary Area (um^2)	37.57 ± 0.50	38.03 ± 0.70	37.72 ± 0.68	37.62 ± 0.60	0.9550
Cortical Porosity (%)	4.44 ± 0.10	4.55 ± 0.10	4.65 ± 0.05	4.37 ± 0.09	0.1331
**10 weeks**					
Whole Body					
BMC (mg)	540.36 ± 8.69[a]	453.71± 8.32[c]	531.14 ± 10.74[ab]	507.70 ± 9.96[b]	**<0.0001**
BMA (cm^2)	10.20 ± 0.28[a]	9.24 ± 0.13[b]	10.32 ± 0.12[a]	9.97 ± 0.16[a]	**<0.0001**
Femur Mid-Diaphysis					
Medullary Area (um^2)	36.76 ± 0.27	37.50 ± 0.39	32.54 ± 3.07	36.90 ± 0.55	0.0752
Cortical Porosity (%)	4.27 ± 0.04	4.50 ± 0.07	4.47 ± 0.12	4.29 ± 0.05	0.0505

Data were analyzed using 1-way ANOVA and are presented as mean ± SE. Within a given row, groups that do not share the same superscript letter are significantly different from each other. Bone mineral content (BMC), bone mineral area (BMA). Sham-operated (SHAM), ovariectomized (OVX), control diet (Con), diet supplemented with dried plum (DP), diet supplemented with crude polyphenol extract (Crude).

Table S2: Simple effects of the PP and CHO components of the crude extract on other whole body DXA and trabecular and cortical microCT parameters.

	OVX-Con	OVX-Crude	OVX-PP	OVX-CHO	*P-Values* PP vs Con	CHO vs Con	PP vs Crude	CHO vs Crude
**5 weeks**								
Whole Body								
BMC (mg)	472.46 ± 7.47	567.27± 11.41	510.31±10.37	531.93 ±7.05	**0.0184**	**<0.0001**	**<0.0001**	**0.0105**
BMA (cm²)	9.72 ± 0.15	10.99 ± 0.19	10.14 ± 0.15	10.53 ± 0.13	0.1759	**0.0003**	**<0.0001**	0.0620
Distal Femur								
TbTh (mm)	0.041 ± 0.001	0.041 ± 0.001	0.041± 0.002	0.042 ± 0.001	0.7550	0.9079	0.7413	0.9215
TbN (1/mm²)	3.09 ± 0.10	3.81 ± 0.04	3.22 ± 0.06	3.71 ± 0.11	0.2509	**<0.0001**	**<0.0001**	0.4110
TbSp (mm)	0.32 ± 0.01	0.26 ± 0.003	0.31 ± 0.007	0.27 ± 0.008	0.2141	**0.0001**	**<0.0001**	0.4130
Lumbar Vertebra								
TbTh (mm)	0.043 ± 0.001	0.048 ± 0.0004	0.046± 0.001	0.047 ± 0.001	**0.0113**	**0.0003**	0.0657	0.3479
TbN (1/mm²)	3.94 ± 0.09	3.95 ± 0.06	3.80 ± 0.08	3.96 ± 0.07	0.2073	0.8652	0.1774	0.9416
TbSp (mm)	0.260 ± 0.01	0.255 ± 0.004	0.267 ± 0.006	0.255 ± 0.005	0.1885	0.8139	0.1385	0.9588
Femur Mid-Diaphysis								
Medullary Area (um²)	38.03 ± 0.70	37.62 ± 0.60	39.17 ± 1.16	39.75 ± 0.79	0.3524	0.1738	0.1963	0.0865
Cortical Porosity (%)	4.55 ± 0.10	4.37 ± 0.09	4.73 ± 0.15	4.57 ± 0.09	0.2669	0.9212	**0.0229**	0.2159
**10 weeks**								
Whole Body								
BMC (mg)	453.71± 8.32	507.70 ± 9.96	499.13 ± 6.25	524.10 ± 5.53	**0.0004**	**<0.0001**	0.4975	0.1727
BMA (cm²)	9.24 ± 0.13	9.97 ± 0.16	9.64 ± 0.05	10.11 ± 0.12	**0.0420**	**<0.0001**	0.1190	0.4633
Distal Femur								
TbTh (mm)	0.045 ± 0.001	0.045 ± 0.001	0.047 ± 0.002	0.043 ± 0.001	0.4481	0.2021	0.4481	0.2427
TbN (1/mm²)	2.81 ± 0.08	3.28 ± 0.08	2.98 ± 0.05	3.53 ± 0.11	0.1936	**<0.0001**	**0.0304**	0.0520
TbSp (mm)	0.36 ± 0.01	0.30 ± 0.01	0.33 ± 0.01	0.28 ± 0.01	0.0673	**<0.0001**	**0.0346**	0.0914
Lumbar Vertebra								
TbTh (mm)	0.047 ±0.0004	0.050 ± 0.001	0.050 ± 0.001	0.050 ± 0.001	**0.0041**	**0.0029**	0.5906	0.4979
TbN (1/mm²)	3.37 ± 0.08	3.88 ± 0.19	3.67 ± 0.04	4.13 ± 0.11	0.1246	**0.0002**	0.2465	0.1460
TbSp	0.30 ± 0.01	0.26 ± 0.01	0.27 ± 0.003	0.24 ± 0.01	0.0721	**0.0001**	0.4567	0.0673
Femur Mid-Diaphysis								
Medullary Area (um²)	37.50 ± 0.39	36.90 ± 0.55	36.43 ± 0.48	36.46 ± 0.39	0.1167	0.0839	0.5020	0.4806
Cortical Porosity (%)	4.50 ± 0.07	4.29 ± 0.05	4.12 ± 0.10	4.36 ± 0.10	**0.0068**	0.2118	0.2337	0.5694

Data are presented as mean ± SE. Bone mineral content (BMC), bone mineral area (BMA). Ovariectomized (OVX), control diet (Con), diet supplemented with crude polyphenol extract (Crude), phenolic enriched diet (PP), and carbohydrate enriched diet (CHO).
